# Attenuation of dopamine‐induced GABA release in problem gamblers

**DOI:** 10.1002/brb3.1239

**Published:** 2019-02-20

**Authors:** Arne Møller, Kristine Rømer Thomsen, David J. Brooks, Kim Mouridsen, Jakob U. Blicher, Kim V. Hansen, Hans C. Lou

**Affiliations:** ^1^ Nuclear Medicine and PET‐Center Aarhus University Hospital Aarhus Denmark; ^2^ Center of Functionally Integrative Neuroscience Aarhus University Aarhus Denmark; ^3^ Center for Alcohol and Drug Research Aarhus University Aarhus Denmark; ^4^ Division of Neuroscience University of Newcastle Tyne UK

**Keywords:** dopamine, GABA, PET, problem gambling, Ro15‐4513, self‐control

## Abstract

**Introduction:**

We have previously shown that an interaction between medial prefrontal and parietal cortices is instrumental in promoting self‐awareness via synchronizing oscillations in the gamma range. The synchronization of these oscillations is modulated by dopamine release. Given that such oscillations result from intermittent GABA stimulation of pyramidal cells, it is of interest to determine whether the dopaminergic system regulates GABA release directly in cortical paralimbic regions. Here, we test the hypothesis that the regulation of the GABA‐ergic system by the dopaminergic system becomes attenuated in problem gamblers resulting in addictive behaviors and impaired self‐awareness.

**Methods:**

[^11^C]Ro15‐4513 PET, a marker of benzodiazepine *α*1/*α*5 receptor availability in the GABA receptor complex, was used to detect changes in synaptic GABA levels after oral doses of 100mg L‐dopa in a double‐blind controlled study of male problem gamblers (*N* = 10) and age‐matched healthy male controls (*N* = 10).

**Results:**

The mean reduction of cortical gray matter GABA/BDZ receptor availability induced by L‐dopa was significantly attenuated in the problem gambling group compared to the healthy control group (*p* = 0.0377).

**Conclusions:**

Our findings demonstrate that: (a) Exogenous dopamine can induce synaptic GABA release in healthy controls. (b) This release is attenuated in frontal cortical areas of males suffering from problem gambling, possibly contributing to their loss of inhibitory control. This suggests that dysfunctional dopamine regulation of GABA release may contribute to problem gambling and gambling disorder.

## 1 INTRODUCTION

In this study, we investigate this hypothesis with ^11^C‐Ro15‐4513 positron emission tomography (PET), a marker of synaptic GABA fluxes, and demonstrate that the dopaminergic activation of GABA release is attenuated in the cerebral cortex of males suffering from problem gambling.

Gambling disorder is highly disabling and characterized by repeated and maladaptive gambling behaviors that persist despite negative personal, social, and financial consequences. Due to its clinical and neurobiological overlap with drug use disorders (Frascella, Potenza, Brown, & Childress, 2010; Grant, Potenza, Weinstein, & Gorelick, 2010; Potenza, 2008), gambling disorder has now been reclassified as a “non‐substance‐related disorder,” in the section on “substance‐related and addictive disorders” in the DSM‐5 (American Psychiatric Association, 2013).

One of the key symptoms in behavioral and drug addiction is problems with self‐control (Ersche et al., 2012; Rømer Thomsen et al., 2013), which is closely related to another hallmark of addiction: diminished self‐awareness, and insight of the affected person into the severity of their disorder (Brevers et al., 2013; Goldstein et al., 2009; Moeller & Goldstein, 2014). Problems related to insight and self‐awareness are major impediments to recovery (Goldstein et al., 2009).

The prefrontal cortex assigns salience to stimuli. Dysfunction of the prefrontal cortex, including the anterior cingulate cortex, plays an important role in drug and nondrug‐related addictions, resulting in problems with self‐control and self‐awareness (Bechara, 2005; Brevers et al., 2013; Changeux & Lou, 2011; Ersche et al., 2012; Goldstein & Volkow, 2011; Posner, Rothbart, Sheese, & Tang, 2007). A diminished ability to recruit prefrontal networks results in a failure of behavior regulation and impaired judgment concerning the salience of stimuli (George & Koob, 2013; Hayashi, Ko, Strafella, & Dagher, 2013). Problems related to self‐control have received most attention and empirical support in gambling disorder and drug addictions, however, problems related to impaired self‐awareness are also considered central and should also receive support (Brevers et al., 2013; Brevers & Noel, 2013; Goldstein et al., 2009; Moeller & Goldstein, 2014).

Previous studies have identified a paralimbic network involved in self‐awareness, involving the medial prefrontal/anterior cingulate cortex, medial parietal/posterior cingulate cortex, thalamus, and striatum (Doering et al., 2012; Lou, Gross, Biermann‐Ruben, Kjaer, & Schnitzler, 2010; Lou, Luber, Stanford, & Lisanby, 2010; Qin & Northoff, 2011). This network has been found to exhibit a bidirectional interaction, with oscillatory activity synchronized throughout a range of beta and gamma frequencies (25–100Hz ) with maximum synchronization at 40Hz  in the gamma range. The network is active at rest, that is, during spontaneous, mainly self‐referential thoughts without intended stimulation. It shows increased activity with increasing degrees of explicit self‐reference (Lou, Gross et al., 2010). Reduced resting functional connectivity between the anterior cingulate and prefrontal cortices has been found in individuals suffering from drug addiction (Ma et al., 2010). Altered paralimbic function which increases susceptibility to reduced self‐control and addictive disorders could develop premorbidly. Alternatively, it could be a consequence of intake of toxic substances. In order to elucidate this question, we have examined levels of gamma synchronization in individuals suffering from gambling disorder with and without a history of drug addiction. In both groups we found decreased synchronization of gamma oscillatory activity between the medial prefrontal/anterior cingulate and medial parietal/posterior cingulate cortices during rest, compared with healthy controls, as well as decreased self‐control measured with the Stop Signal task (Rømer Thomsen et al., 2013). Hence, reduced self‐control in gambling disorder is linked to decreased paralimbic interaction.

This paralimbic network is regulated by dopamine and other neurotransmitters via fast spiking parvalbuminergic GABA interneurons (Changeux & Lou, 2011; Lou, Changeux, Changeux, & Rosenstand, 2016). These interneurons act as a natural “mini brain,” balancing a wide spectrum of neurotransmitters in the regulation of pyramidal cell activity (Joensson et al., 2015; Lou, Joensson, Biermann‐Ruben et al., 2011; Lou, Joensson, & Kringelbach, 2011; Lou, Skewes et al., 2011). The interaction between dopamine and GABAergic function posited to promote self‐awareness has primarily been inferred from studies on the activity of subcortical regions in rodents. These studies were only indirectly relevant to self‐awareness and conscious self‐control. Using a combination of an oral L‐dopa challenge to exogenously raise brain dopamine and measurements of GABA_A_/BDZ receptor availability with [^11^C]Ro15‐4513 PET, we obtained evidence that rises in synaptic dopamine activated GABA neurotransmission and enhanced gamma synchronization and self‐awareness in humans. The L‐dopa‐induced increases in GABA release result in increased GABA receptor occupancy predominantly in the medial prefrontal/anterior cingulate region crucial for self‐awareness (Joensson et al., 2015; Lou, Rosenstand et al., 2016). GABA‐A receptors and alpha‐1 subunits are ubiquitous in gray matter. Αlpha 5 subunits, however, are particularly found in limbic cortical areas. Synchronized oscillations in the gamma range are linked to cognitive function (Fuentemilla, Palombo, & Levine, 2018; Schnitzler & Gross, 2005), and causality can be inferred from the electrophysiological findings of Lou et al. (2004) and Luber, Lou, Keenan, and Lisanby (2012). We have previously shown that synchronization of gamma oscillatory activity between medial prefrontal and parietal regions is reduced in individuals suffering from gambling disorder (Rømer Thomsen et al., 2013). This synchronization is known to facilitate self‐awareness (Lou, Changeux et al., 2016) and its reduction is associated with impaired self‐awareness and may also be instrumental in the development of an addiction to gambling and allied problems. Levels of synaptic GABA are regulated by dopamine release and it is GABA activation that leads to the periodic inhibition of pyramidal cells that results in synchronization of oscillatory activity across brain regions (Lou, Rosenstand et al., 2016). We, therefore, propose that attenuation of the normal dopamine regulation of GABA neurotransmission may underlie impaired self‐control and self‐awareness, both of which are characteristically present in individuals suffering from gambling disorder.

Here, we hypothesize that dopaminergic activation of GABA neurotransmission becomes attenuated in males suffering from problem gambling, as a manifestation of problems related to their self‐control and self‐awareness. We tested the hypothesis by challenging participants with oral L‐dopa to increase their brain dopamine and used [^11^C]Ro15‐4513 PET, a marker of GABA_A_/BDZ receptor availability, to detect increases in synaptic GABA levels. A number of recent PET‐studies using the [^11^C]Ro15‐4513 ligand point to a prominent role of altered GABA neurotransmission in individuals addicted to alcohol (Lingford‐Hughes et al., 2012), opiates (Lingford‐Hughes et al., 2016), nicotine (Stokes et al., 2013), and gambling (Mick et al., 2016). To our knowledge, our study is the first to directly examine altered GABA regulation by exogenous dopamine as evidenced by GABA‐A receptor availbility in a nondrug addictive disorder. Furthermore, by focusing on a well‐defined group with a behavioral addiction, we were able to study possible alterations in dopamine regulation of GABA neurotransmission independently of any toxic effects of psychotropic drugs.

## 2 MATERIALS AND METHODS

### 2.1 Study subjects

We selected two groups of participants: A group of male participants suffering from, or with a recent history of, problem gambling and an aged‐matched male control group. The study was limited to male participants in order to avoid different stages of the menstrual cycle as a confound. Furthermore, in healthy samples, women have been shown to differ significantly from men in their cortical dopamine transmission and D2 receptor availability (Love et al., 2012). This focus on males is in line with the overrepresentation of males among individuals suffering from gambling disorder (Kessler et al., 2008).

Participants suffering from problem gambling (PG) were recruited from the Aarhus and Odense *Centre for Gambling Disorder*, which is one of the largest treatment facilities for individuals suffering from gambling disorder in Denmark. The criteria for admission to the treatment center, and into the present study, was confirmatory responses on seven out of a total of 20 questions in a questionnaire developed by Gamblers Anonymous to assess the severity of problem gambling. This self‐report measure has shown good psychometric properties, including high reliability and good convergent validity. Ratings have a high correlation with the South Oaks Gambling Screen (Ursua & Uribelarrea, 1998). The questions focus on difficulties in keeping a job, family problems, gambling with the purpose of paying back debt, sleep problems, and criminal offenses attributed to gambling. These symptoms overlap with the DSM‐5 criteria for gambling disorder (American Psychiatric Association, 2013), however, because a formal diagnosis was not given to our participants, we refer to the included participants as problem gamblers. Healthy controls were recruited from a database of individuals who were interested to participate in research studies. The PG participants were included in the study if they were currently, or had a recent history of, receiving treatment for gambling disorder, had responded positively to a minimum of seven out of 20 questions in a validated measure of problem gambling (Ursua & Uribelarrea, 1998), and did not suffer from a neurological or other psychiatric disorder. Two of the PG participants had a history of drug addiction (primarily stimulants) 5 and 12years  ago when assessed with the Mini International Neuropsychiatric Inventory (Lecrubier et al., 1997). The healthy controls were included in the study if they did not suffer from any psychiatric or neurological disorder. None of the healthy controls had a history of drug addiction. None of the PG or healthy controls received cerebrally active medication. Four out of 10 of the healthy controls and 5 out of 10 of the PG participants were currently smoking tobacco. In a previous study (Rømer Thomsen et al., 2013) involving healthy controls and individuals suffering from gambling disorder, smoking was not found to influence the prevalence of abnormal gamma oscillations. The focus of the present study was to identify the pathogenesis of such oscillations and we did not exclude smokers. Ten male PG participants (mean age: 32.4; *SD* 3.7) and 10 healthy controls (mean age: 30.8; *SD* 2.2) were included in the study. An independent *t* test showed no difference in mean age between the two groups, *t*(18) = 0.3708, *p* = 0.1305.

The study was approved by the local ethics committee (*De Videnskabsetiske Komitéer for Region Midtjylland*) and was conducted according to the Declaration of Helsinki. Participants received oral and written information about the study and gave their written consent before participating in the study.

### 2.2 Study design

Each participant was studied on two separate days in a counterbalanced order (with an interval of several days). Before PET scanning participants received a L‐dopa challenge or placebo orally in identical capsules containing either Sinemet (MSD, 100mg  L‐dopa plus 25mg  Carbidopa to block peripheral metabolism of L‐dopa, thereby increasing tracer delivery to the brain) or placebo (Starch). Participants and researchers responsible for PET injection and calculation of [^11^C]Ro15‐4513 binding potentials were all blinded. L‐dopa or placebo was administered 30–45min  before the intravenous injection of 400 (range 375–425) MBq [^11^C]Ro15‐4513 through an antecubital vein in 10ml  of normal saline over 30s. The 30–45min  interval is based on the timing of peak plasma concentrations and onset of clinical relief in Parkinson's disease after oral Sinemet medication (Joensson et al., 2015). The order in which participants were studied was randomized.

### 2.3 MRI and PET imaging

#### 2.3.1 Magnetic resonance imaging (MRI)

All participants were scanned with 3T MRI for coregistration with PET (Siemens Trio, Erlangen, Germany). A T1 MPRAGE scan (TR/TE 2,420/3.7ms , 1 mm  isotropic resolution, scan time 5½ min) was performed.

#### 2.3.2 PET

[^11^C]Ro15‐4513 was synthesized by adapting the procedure of Halldin, Farde, Litton, Hall, and Sedvall (1992). Binding of the PET ligand [^11^C]Ro15‐4513 is sensitive to interstitial GABA levels evidenced as a fall in availability of α1 subtype sites on the GABA complex available for occupancy (Semyanov, Walker, Kullmann, & Silver, 2004; Stokes et al., 2014). While [^11^C]Ro15‐4513 binds to both the α1 and α5 receptor subtypes, the α1 subtype is present at a greater density and increases in brain GABA have been shown to reduce its availability, while α5 binding site availability is little changed. In the present study, it was not possible to kinetically separate the signals arising from α1 and α5 subunit binding in the GABA complex due to the lack of an arterial [^11^C]Ro15‐4513 input function. Here, we have assumed that any decreased Ro15‐4513 binding seen after L‐dopa administration represents competitive occupancy of α1 sites by endogenous GABA (Lingford‐Hughes et al., 2002).

Participants were placed in the scanner with their orbitomeatal line parallel to the transaxial plane of the tomograph. Head position was monitored via laser crosshairs and a video camera. In order to correct for attenuation of emitted radiation by skull and tissues, a transmission scan was acquired using a single rotating photon point source of 150MBq  of ^137^Cs. Three‐dimensional PET was acquired over 60min  using an ECAT EXACT HR++ (CTI/Siemens 966; Siemens PET/CT Biograph, Erlangen, Germany) camera, which covers an axial field of view of 23.4cm  and provides 95 transaxial planes. The tomograph has a spatial resolution of 4.8 + 0.2mm  FWHM (transaxial, 1cm  off axis) and 5.6mm + 0.5mm  (axial, on axis) after image reconstruction. PET and MRI were coregistered to a common MR T1 atlas from Montreal Neurological Institute (MNI) (Collins et al., 1998) using PMOD (PMOD Technologies Ltd., Zurich, Switzerland). The kinetic parameters of [^11^C]Ro15‐4513 were determined by the SRTM2 method using cerebellum as reference for nonspecific binding (Wu & Carson, 2002). No FDR or cluster corrections were used as the regions were predefined. Before having PET in a room with subdued light, the participants were asked to lie still with their eyes closed.

### 2.4 Statistical analysis

In order to test if dopamine regulation of GABA release was the same in the two groups, we performed a mixed‐model analysis with treatment (L‐dopa challenge or placebo) and group (PG participants or healthy controls) as fixed effects and person as a random effect.

## 3 RESULTS

**Figure 1:**
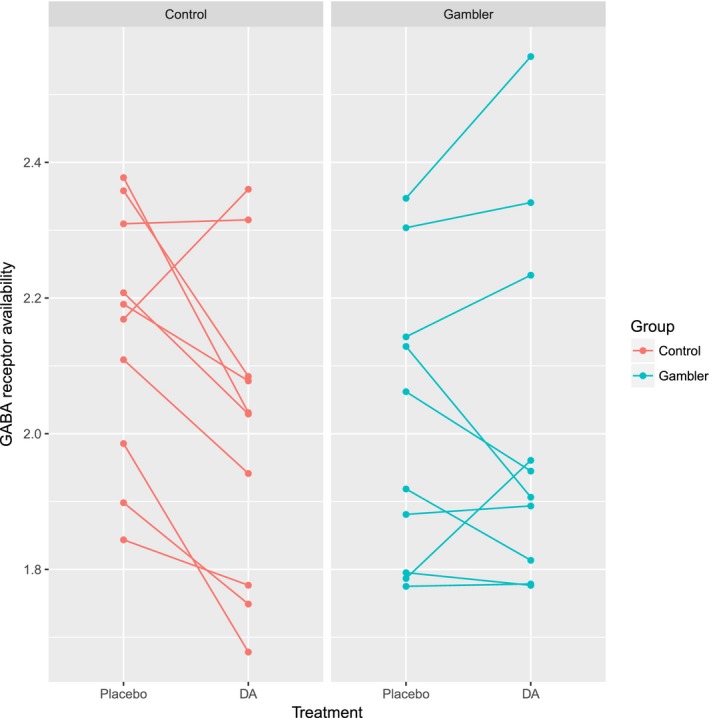
Among healthy controls, GABA receptor availability in total gray matter is generally decreased with L‐dopa. This trend is absent in 70% of the individuals suffering from problem gambling

Mean GABA‐A receptor availability was significantly reduced by the L‐dopa challenge in the healthy control group compared to the PG group (*p* = 0.0377, Table 1). Individually, cortical gray matter GABA‐A receptor availability was decreased after L‐dopa in eight out of 10 healthy controls but only three out of 10 PG participants (see Figure 1). This implies that GABA release is induced by exogenous dopamine in a majority of healthy controls leading to a reduction in their GABA receptor availability and this reduction is attenuated in individuals manifesting problem gambling.

**Table 1:** Dopamine regulation of GABA release in gray matter

	Value	Std. Error	DF	*t*‐value	*p*‐value
(Intercept)	2.1449724	0.07	18	30.172879	0.0000
Group (PG^a^, HC^b^)	−0.1308278	0.10	18	−1.301308	0.2096
Treatment (L‐dopa, placebo)	−0.1405559	0.05	18	−3.035623	0.0071
Group:Treatment	0.1469332	0.07	18	2.243901	0.0377

a Individuals suffering from problem gambling.

b Healthy controls.

**Figure 2:**
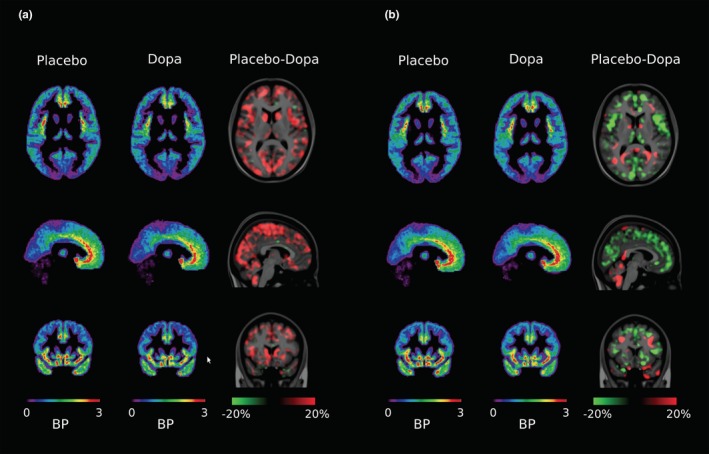
(a)Distribution of the effect of dopaminergic activation of GABA release demonstrated by reduced GABA‐A/BDZ receptor availability in healthy controls. This effect was prominent in prefrontal regions and insula and present throughout neocortical regions and cerebellum. (b) In problem gambling this effect of L‐dopa was generally attenuated or even reversed in most of neocortex

Figure 2 illustrates the distribution of GABA‐A receptor binding of ^11^C‐ Ro15‐4513. In Figure 2a (healthy controls), there is a global decrease in ligand binding throughout the brain after L‐dopa administration. This indicates decreased availability of binding sites due to increased endogenous GABA release after exogenous dopamine stimulation. Figure 2b shows that the effect of L‐dopa on decreasing receptor availability is attenuated in the neocortex and limbic cortex of individuals suffering from problem gambling. Interestingly normal L‐dopa stimulation of GABA release was seen in the brain stem of these gamblers.

## 4 DISCUSSION

In line with our hypothesis, we found significantly reduced dopaminergic activation of cortical gray matter GABA neurotransmission in PG participants compared to healthy controls. This was evidenced by decreased ^11^C‐Ro15‐4513 binding to GABA‐A/BDZ sites induced by L‐dopa administration to healthy controls which was significantly attenuated in the PG group. This implies that synaptic GABA release is induced by dopamine rises in healthy controls, and that this effect is reduced or even reversed in individuals suffering from problem gambling.

As reported previously (Lou, Rosenstand et al., 2016), exogenous dopamine activates GABA neurotransmission in healthy controls. After placebo, highest [^11^C]Ro15‐4513 BDZ ligand binding was seen in medial prefrontal/anterior cingulate cortex and left and right insula. After dopamine challenge, cortical ligand binding was reduced in eight of 10 healthy controls. Thus, the distribution of the stimulating effect of dopamine occurs broadly in neocortex in healthy controls. Here we show, that this effect is reduced and, occasionally, inverted in the neocortex in individuals suffering from problem gambling. The present results therefore support our theory of malfunctioning of dopamine regulation of GABA function in males suffering from problem gambling.

A prominent role of dysfunctional GABA neurotransmission in addiction has been suggested by recent studies with [^11^C]Ro15‐4513 PET in individuals suffering from addiction. Alcohol (Lingford‐Hughes et al., 2012) and opiate (Lingford‐Hughes et al., 2016) addiction were linked to lower levels of GABA‐A receptor availability in limbic regions, compared to controls, while gambling addiction (Mick et al., 2016) and a history of cigarette smoking (Stokes et al., 2013) were associated with higher levels of limbic GABA‐A receptor availability compared to controls.

Hoerbelt, Lindsley, and Fleck (2015) have demonstrated that the dopaminergic system regulates GABA receptors directly in the striatum using the patch‐clamp technique. In our previous report (Lou, Rosenstand et al., 2016) we showed that dopaminergic activation of GABA release occurs directly throughout the paralimbic cortex, in particular in the medial prefrontal cortex. This binding is compromised in individuals suffering from problem gambling—as shown here. The direct dopaminergic activation of GABA release brings the physiological properties of the GABA‐A receptor molecules into focus. The GABA‐A receptor comprises an ion channel, organized as a pore between five protein subunit complexes. The pore allows passage of chloride ions, and, hence, electrical pulses when stabilized in an open conformation. Such stabilization occurs by binding of GABA to a site on the complex. The binding of GABA to this receptor is not specific. The affinity of physiological, including dopamine, and foreign molecules depends on the protein composition of the five pentameric molecules constituting the pore. The GABA receptor is therefore a vehicle for direct dopamine regulation of GABA neurotransmission. Alternatively dopamine may influence GABA transmission indirectly acting via D2 auto‐ and postsynaptic receptors. Of interest, here is the discovery from reviewing available data that the subtype composition of the pentameric GABA‐A pore is abnormal in addiction (Stephens, King, Lambert, Belelli, & Duka, 2017). This finding may provide a mechanism for the disturbance of dopamine regulation of GABA neurotransmission in problem gambling.

Some limitations in this study must be acknowledged. First, because of the lack of a formal diagnosis, we cannot be sure that the participants recruited from the Gambling Disorder Treatment Facility fulfilled DSM‐5 criteria for gambling disorder, and hence we have referred to them as problem gamblers. Second, the study only included men (in order to avoid different stages in the menstrual cycle as a confound; and because healthy women have been shown to differ significantly from men in cortical dopamine transmission and D2 receptor availability), and hence our findings cannot be directly generalized to women. Third, because of the small sample size, it was difficult to statistically control for scan order (the small sample size was a necessity due to the two scan design and the fact that PET is expensive).

This study takes a first step in directly examining interactions between the dopamine and GABA systems in an addictive disorder: males suffering from problem gambling. Future studies with larger sample sizes are needed to replicate and extend these findings, preferably in samples of individuals suffering from gambling disorder and different types of drug addictions, and by including measures of self‐control and self‐awareness, as well as more precise descriptions of lifestyle (including smoking). Potentially, these findings may have clinical implications by informing future pharmacological studies testing the effect of GABA‐enhancing medications in gambling disorder. For example, one could speculate that reduced GABA release after dopamine‐enhancing drugs or endogenous dopamine release in problem gamblers leads to disinhibition of their urges to gamble and that GABA agonists such as benzodiazepines are likely to suppress these urges.
